# Mitomycin C reduces abundance of replication forks but not rates of fork progression in primary and transformed human cells

**DOI:** 10.18632/oncoscience.70

**Published:** 2014-07-27

**Authors:** Keffy Kehrli, Julia M. Sidorova

**Affiliations:** ^1^ Department of Pathology, University of Washington, Seattle, WA

**Keywords:** DNA replication, cell cycle, S phase, mitomycin C, DNA crosslinks, Fanconi Anemia protein D2, DNA fiber analysis

## Abstract

DNA crosslinks can block replication *in vitro* and slow down S phase progression *in vivo*. We characterized the effect of mitomycin C crosslinker on S phase globally and on individual replication forks in wild type and FANCD2-deficient human cells. FANCD2 is critical to crosslink repair, and is also implicated in facilitating DNA replication. We used DNA fiber analysis to demonstrate persistent reduction in abundance but not progression rate of replication forks during an S phase of MMC-treated cells. FANCD2 deficiency did not eliminate this phenotype. Immunoprecipitation of EdU-labeled DNA indicated that replication was not suppressed in the domains that were undergoing response to MMC as marked by the presence of γH2AX, and in fact γH2AX was overrepresented on DNA that had replicated immediately after MMC in wild type through less so in FANCD2-depleted cells. FANCD2-depleted cells also produced fewer tracks of uninterrupted replication of up to 240Kb long, regardless of MMC treatment. Overall, the data suggest that crosslinks may not pose a block to S phase as a whole, but instead profoundly change its progress by reducing density of replication forks and causing at least a fraction of forks to operate within a DNA damage response-altered chromatin.

## INTRODUCTION

Cellular response to DNA crosslinks involves more than one DNA repair and checkpoint pathway, making these lesions a likely challenge to cancer cells that have either lost or deregulated at least some of the DNA damage response machineries [1]. Crosslinker drugs such as CisPt, nitrogen mustards, mitomycin C (MMC) and others, generate a mixture of intra-and interstrand links as DNA adducts, which are capable of blocking DNA polymerases in a test tube. Interstrand crosslinks (comprising 1-10% of crosslinks) are considered the most cytotoxic. *In vivo*, slowing of S phase progression is a known phenotype of crosslink damage, often explained in terms of replication fork blockage.

The interaction between DNA replication and crosslinks (interstrand crosslinks in particular) has been a subject of intense investigation. Crosslink repair does not require replication but is facilitated by it [2-5]. Early models proposed that replication forks stalled at crosslinks, and this enabled their recognition and repair (for review [6, 7]). Studies in Xenopus extracts led to a model where two forks converge on a crosslink, followed by repair [8, 9]. On the other hand, recent evidence suggests that bulky adducts such as may arise from interstrand crosslink unhooking can be bypassed by both replisome and replicative helicase if they are on the lagging strand [10], and moreover, *in vivo*, replication forks will transiently slow and then bypass at least some types of crosslinks (trioxalen/UVA) by repriming downstream of them [11]. Thus, as with other kinds of damage, genomic replication as a whole may be altered by crosslinks rather than blocked, through a combination of “filling in” around lesions or bypassing them.

Fanconi Anemia (FA) is a human heritable syndrome characterized by developmental defects, bone marrow failure and cancer predisposition. FA is caused by mutations in a higher eukaryote-specific network of genes involved in repair of DNA crosslinks and formaldehyde-induced damage, likely including DNA-protein crosslinks. There are sixteen FA genes identified thus far; many of them have functions outside recognition and repair of crosslinks [12, 13]. FANCD2 is one of such genes. FANCD2 plays a crucial role in crosslink repair as a part of the FANCD2/FANCI complex that is recruited to a crosslink early in the recognition process to facilitate checkpoint signaling [4], as well as DNA strand incision, and subsequent steps of repair [9]. FANCD2 is also a part of a complex detected on unreplicated or unresolved regions of DNA in late S/G2 phases of the cell cycle [14, 15].

FANCD2 has also been implicated in supporting DNA replication. Loading of CDC45 onto chromatin is reduced without FANCD2, and interorigin distance is increased [16], suggesting a role in replication initiation. During fork stalling caused by ribonucleotide depletion or inhibition of replicative DNA polymerases, FANCD2 is important to limit resection of nascent DNA by facilitating loading of RAD51 [17, 18]. FANCD2 also cooperates with BLM to promote fork restart after stalling [18], and interacts with MCM helicase to restrain the helicase and/ or replisome from generating partially single-stranded DNA under semi-permissive conditions of replication during partial ribonucleotide depletion [19]. At present it is unclear whether these are facets of one and the same function.

We were interested in characterizing the effect of acute mitomycin C (MMC) crosslinker treatment on an ongoing S phase genome-wide and on individual replication forks, and whether or not it is affected by FANCD2 deficiency. MMC forms helix-distorting intra and interstrand crosslinks between guanine residues [20]. Cells treated with MMC generate comparatively less ssDNA than UV and HU, but are nevertheless able to activate robust S phase checkpoint by a mechanism involving FANCM/FAAP24 [21], the protein also needed for crosslink bypass [11]. We used high resolution techniques to inspect S phase progression after MMC and here we report three key findings. First, addition of MMC to S phase cells rapidly triggers a 40-50% reduction in abundance of replication forks which can persist for hours after the drug, while global fork progression rate is not affected. This response is the same in primary and transformed cells and is not significantly modified by FANCD2 deficiency. Second, DNA replication that occurs after MMC is not excluded from genomic domains undergoing DNA damage response (DDR) as judged by presence of γH2AX. γH2AX is overrepresented on newly-replicated DNA after MMC, and less so in FANCD2-deficient cells. Third, FANCD2 depletion reduces the prevalence of long, uninterrupted segments of DNA replication, regardless of MMC treatment.

## RESULTS

### Cellular response to a pulse-treatment with MMC during S phase

**Figure 1:**
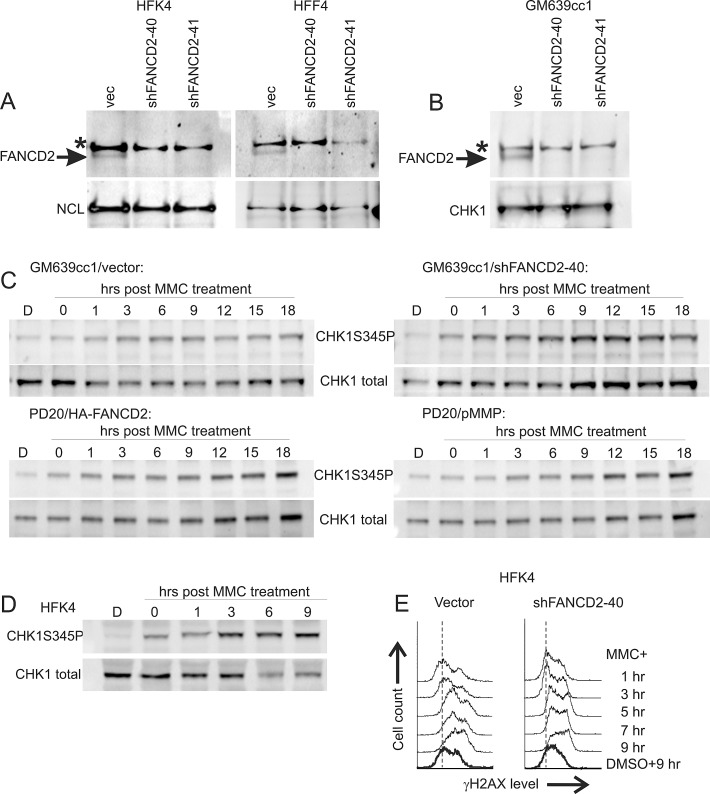
Characterization of response to MMC in human fibroblasts and keratinocytes with and without FANCD2 deficiency **A**) Western blots of FANCD2 expression in isogenic primary human keratinocytes HFK4 and fibroblasts HFF4 infected with an empty lentiviral vector pLKO.1 (vec) or vectors expressing two different shRNAs against FANCD2. **B**) A Western blot of GM639cc1 SV40 fibroblasts infected with the same lentiviral constructs. NCL, nucleolin, or CHK1 were used as internal controls. An asterisk denotes a nonspecific band typical of the antibody used. **C**) Western blots visualizing phospho-CHK1 (Serine 345-phosphorylated) and total CHK1 in GM639cc1 cells with and without FANCD2 depletion and in PD20 *fancd2−/−* fibroblasts with and without HA-FANCD2 complementation. pMMP is an empty vector used to stably transfect PD20 *fancd2−/−* fibroblasts as a control. Cells were allowed to recover for up to 18 hrs after MMC treatment (25μM/1hr). **D**) A Western blot of HFK4 cells treated with 25μM of MMC for 1hr and allowed to recover for indicated times before harvest. D: DMSO (vehicle) treated control. Serine 345-phosphorylated (CHK1S345P) and total CHK1 were visualized. E) Flow cytometry profiles of control (vector) or FANCD2-depleted HFK4 cells recovering from MMC (25μM /1hr) for indicated times, and DMSO-treated controls. Cells were stained for γH2AX expression.

We used SV40-transformed Fanconi Anemia patient-derived FANCD2-deficient PD20 fibroblasts with or without complementation by His-tagged FANCD2; unrelated SV40-transformed fibroblasts GM639cc1, or primary fibroblasts (HFF4) and keratinocytes (HFK4) derived from the same healthy donor and depleted of FANCD2 (Figures 1A,B, S1A). FANCD2 depletion in primary cells depressed their growth (Figure S1D, E) and reduced S phase population in fibroblasts though not in keratinocytes (data not shown). We used a short, high-dose MMC treatment regimen (25μM/1hr) in order to follow a homogeneous subpopulation of S phase cells as they progress in a synchronous manner through the stages of the response. We verified that this MMC regimen was cytotoxic and induced checkpoint responses. Specifically, significant cell death was detectable at 72 hrs after the drug (Figure S1B, C) and sustained phosphorylation of CHK1 on S345 (Figure 1C, D) and CHK2 on T68 (Figure S2), was detectable within the first hours after MMC. By flow cytometry, accumulation of γH2AX was evident in MMC-treated S phase (EdU+) and non-S phase (EdU-) primary cells for up to 9 hrs after the drug (Figures 1E, S3A, B). As expected, EdU+ cells treated with MMC slowed progression of their S phase (Figure S3A). Overall, in all cell types studied, cytotoxicity as well as induction of CHK2 T68P, CHK1 S345P, and γH2AX presented evidence of active S phase and DNA damage checkpoint response to the MMC treatment regimen used.

### A modest effect on BrdU incorporation up to 8 hrs after MMC

**Figure 2:**
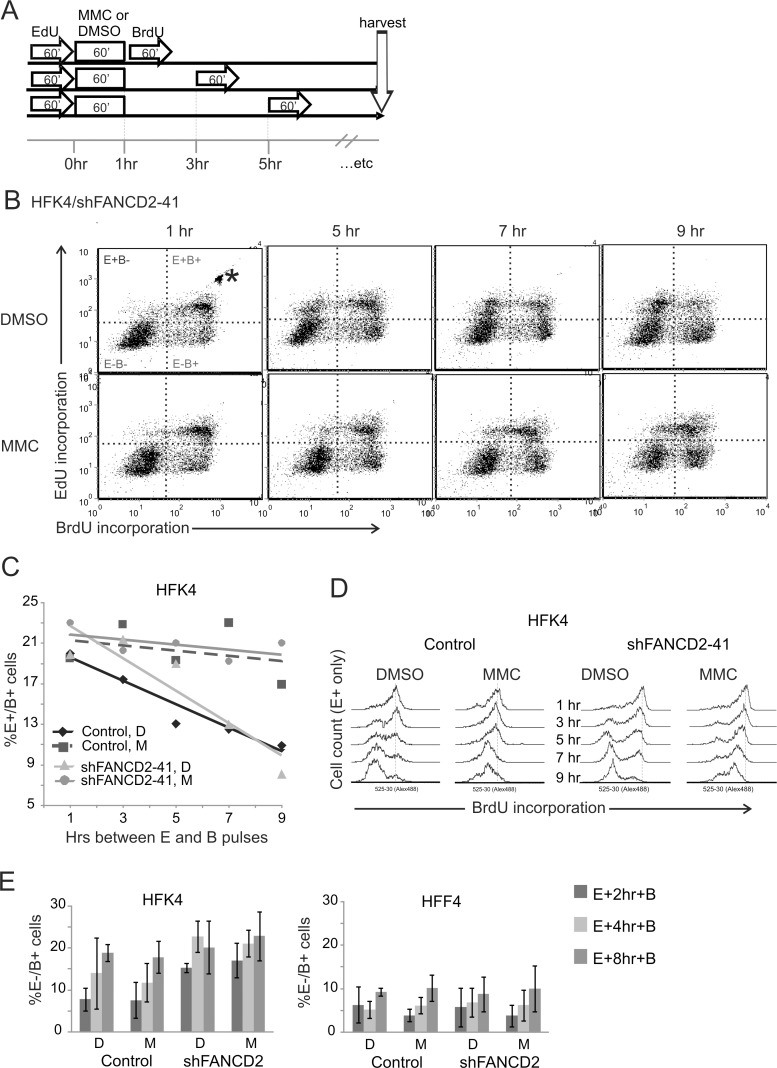
Flow-cytometric analysis of replication after MMC reveals mild replication inhibition in primary human cells **A**) An experimental design. Cells were pulse-labeled with EdU (60 min), treated with MMC (25μM /1hr) or vehicle (DMSO), and allowed to recover, then pulse-labeled with BrdU (60 min). Note that time stamps used throughout this Figure refer to the interval between the end of EdU pulse and the start of BrdU pulse, as shown in (A). All samples were harvested at the same time at the end of the experiment. **B**) Example of flow-cytometric data. Shown are dot plots of HFK4 cells depleted of FANCD2. Cells were stained for EdU (Y axis) and BrdU (X axis). Dotted lines mark the quadrants for positions of four possible cell populations as defined by their EdU and BrdU staining levels. EdU+/BrdU+ cells (upper right quadrant) are cells that were treated by MMC (or DMSO) in an ongoing S phase and that continue to replicate DNA after MMC. EdU-/BrdU+ cells (lower right quadrant) are cells that were treated by MMC (or DMSO) outside of S phase and are entering S phase after MMC. EdU+/BrdU-cells (upper left quadrant) are cells that were treated in S phase and have now exited S phase or stopped replicating. An asterisk in an upper left panel marks position of residual calibration beads. **C**) Declines in percentages of EdU+/BrdU+ (ongoing S phase) cells over time were derived from dot plots as in (B) and plotted. X axis represents hours lapsed between EdU and BrdU pulses. Lines represent trendlines derived from the data. Shown is a representative experiment performed with HFK4 cells. **D**) Flow cytometry profiles of BrdU incorporation levels in EdU+ HFK4 cells from a representative experiment. Indicated times are hours lapsed between EdU and BrdU pulses. **E**) Percentages of EdU-/BrdU+ (new S phase) cells at indicated times between pulses after MMC (or DMSO) treatment were derived from dot plots as in (B) and plotted as bar graphs. Two independent experiments performed with control and FANCD2-depleted, HFF4 and HFK4 cells were averaged. Controls are non-specific shRNA (shNS) expressing cells for HFF4s and empty vector-expressing cells for HFK4s. Error bars are standard deviations.

Next, we used flow cytometry to track S phase progression before and up to 8 hrs after MMC. These experiments were performed with primary isogenic fibroblasts and keratinocytes. We pulse-labeled cells with EdU prior to MMC or vehicle, DMSO, treatment, and with BrdU at several time points after it (Figure 2A). Cells were stained for EdU and BrdU incorporation as shown in Figure 2B, and the data were quantified (Figure 2C-E). We detected no reduction in BrdU incorporation after MMC compared to contemporaneous vehicle-treated samples for the first 5 hrs (Figure 2B-D). 7 hrs after MMC and later, vehicle-treated cells that were in S phase at the time of treatment (EdU+ cells), began to exit S phase and therefore stopped incorporating BrdU (Figure 2C, D). MMC-treated EdU+ cells began to slowly reduce BrdU incorporation (Figure 2C, D). Importantly, for up to 9 hrs these untransformed, low-passage cells continued to incorporate label into DNA regardless of MMC treatment or FANCD2 status.

We also observed entry of new cells into S phase (appearance of EdU-/BrdU+ population), both in control and FANCD2-depleted cell lines and regardless of MMC treatment (Figure 2B, E). In keratinocytes, by the end of the time course new EdU-/BrdU+ cells reached 20% of all cells (Figure 2E, left panel), becoming as abundant as EdU+ cells. In isogenic fibroblasts, EdU-/BrdU+ cells accumulated only up to 10% of all cells (Figure 2E, right panel), which was a third as abundant as EdU+ cells.

### Reduced abundance of replication forks after MMC in primary or transformed human cells

**Figure 3:**
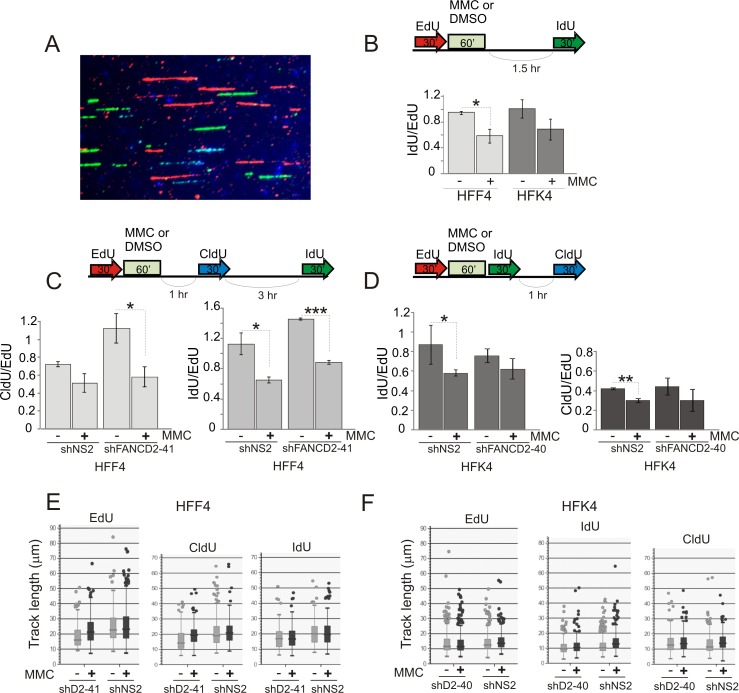
maRTA analysis reveals that MMC treatment results in persistent reduction of fork abundance in primary cells **A**) An example of data generated using labeling of cells with three different analogs, and staining for each label. CldU, blue; IdU, green; and EdU, red. The image is from the set generated with HFK4 cells expressing non-specific shRNA (shNS2). **B**) An experimental design and results obtained with isogenic HFF4 and HFK4 cells. Cells were pulse-labeled with EdU followed by 25μM MMC (or DMSO) pulse, and allowed to recover for 1.5 hrs followed by a pulse of IdU. EdU and IdU replication tracks were counted as described in Materials and Methods in two replicate samples to derive ratios of track numbers (IdU to EdU), which were then averaged and plotted. Here and elsewhere in this Figure, statistical significance was determined in t tests. * denotes p values ≤0.05; **= p≤0.005, etc. Absence of an asterisk indicates that the difference did not reach statistical significance. Error bars here and elsewhere are standard deviations. Average total track number per sample was 896 (min=552, max=1296). **C, D**) Experimental designs and results obtained with HFF4 and HFK4 cells expressing non-specific shRNA or shRNA against FANCD2. Data analysis was as in (B) above. The data represent averages of two replicate samples. Average numbers of tracks counted per sample are 746 (min=341, max=964) for HFF4 and 918 (min=495, max=1521) for HFK4. **E, F**) EdU, CldU, and IdU track lengths from sets counted in (E, F) above are plotted as box plots.

**Figure 4:**
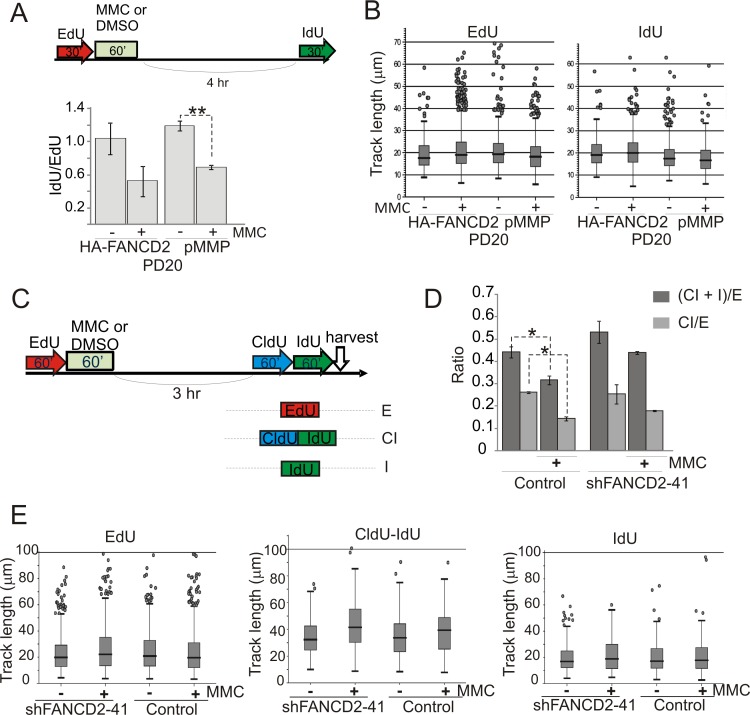
maRTA analysis reveals that MMC treatment results in persistent reduction of relative fork abundance in transformed cells **A**) An experimental design and results obtained with PD20 cells stably expressing HA-FANCD2 transgene and PD20 controls (with an integrated empty pMMP vector). Cells were pulse-labeled with EdU followed by 25μM MMC (or DMSO) pulse, and allowed to recover for 4 hrs followed by a pulse of IdU. EdU and IdU replication tracks were counted as described in the legend to Figure 3 to derive ratios of track numbers (IdU to EdU), which were then averaged and plotted. Here and elsewhere in this Figure statistical significance was determined in t tests. * denotes p values ≤0.05; **= p≤0.005, etc. Absence of an asterisk indicates that the difference did not reach statistical significance. Average total number of tracks counted per sample was 649 (min=316, max=945). Error bars are standard deviations. **B**) Lengths of EdU and IdU tracks in the samples described above were plotted as box plots. **C**) An experimental design and types of tracks generated with this design using GM639cc1 cells expressing empty vector (pLKO.1) or FANCD2 shRNAs. **D**) Quantitation of data generated with the design shown in (C). Numbers of EdU, CldU-IdU, and IdU tracks were counted in two replicate sets of samples. Mutual ratios of indicated track types were averaged and plotted. Error bars are standard deviations. An average total of 690 (min=463, max=1121) tracks were counted per sample. **E**) Lengths of indicated track types from experiments shown in (C, D) were plotted as box plots.

To better visualize the nature of ongoing DNA replication after MMC, we used maRTA (microfluidic-assisted Replication Track Analysis) on cells labeled before and after MMC within the same S phase, similar to the time course used for FACS analyses in Figure 2. These experiments were performed on primary human fibroblasts and keratinocytes (Figure 3) as well as on transformed fibroblasts (Figure 4). Each sample was pulse-labeled with EdU prior to MMC (or vehicle), and with other nucleotide analogs (CldU and/or IdU) at various times after the drug. The choice of time points (Figure 3A-D) was dictated by the need to minimize the fraction of new S phase cells (e.g. EdU-/IdU+) in samples used for maRTA. Thus, the gap between EdU and CldU or IdU pulses was kept short for keratinocytes (Figure 3B, D). The isolated DNA contains non-contiguous tracks of one of the three colors, and their relative frequencies give an estimate of fork abundance before and after MMC (Figure 3A).

In all cases we were able to observe anywhere between a 25 to 50% drop in relative abundance of tracks labeled 1.5 to 4.5 hrs after MMC when compared to contemporaneous untreated samples. This drop was also present in FANCD2-depleted primary fibroblasts. In primary keratinocytes, the drop was less pronounced overall and did not reach statistical significance for FANCD2-depleted samples. Particularly for keratinocytes, since analysis samples may still contain a contamination by cells that newly entered S phase (i.e. EdU-/IdU+ and EdU-/CldU+ cells), the observed value (25-50%) by which MMC decreases the number of ongoing forks may be an underestimate. Based on the observed level of new S phase contamination (e.g. up to 20% in keratinocytes, Figure 2E, left panel) it can be assumed to be up to 20% higher, i.e. between 40 and 60%. Overall, these results suggest a decrease in a number of forks within the ongoing S phase upon MMC treatment.

We found no reduction in the rate of fork progression after MMC. In each cell type, the length distributions of tracks labeled before, after MMC, or without MMC, were essentially the same, even though these lengths differed between cell types, e.g. primary keratinocytes displayed up to two-fold shorter tracks than the isogenic primary fibroblasts (Figure 3E, F). The latter observation may point to cell type-specific differences in replication parameters.

The partial though persistent reduction in fork abundance but not fork rate after MMC was also observed in non-isogenic transformed fibroblasts (Figure 4), suggesting that this effect was general and was not altered by transformation status. In addition, mutation in FANCD2 did not eliminate MMC-induced reduction in fork abundance (Figure 4A). Finally, long, uninterrupted tracks that were extended for up to 120 min, exhibited the same reduction in abundance and not length, as shorter tracks (Figure 4C-E). For example, in MMC-treated GM639cc1 cells, CI tracks became about two-times less abundant relative to E tracks. FANCD2 depletion reduced but did not eliminate this effect. As a whole, the results suggest that despite the global effect on fork abundance, MMC, unlike UV, X-rays, or CPT, does not appear to cause a global reduction of fork elongation rate (see a direct comparison between CPT and MMC in Figure S4).

The likely interpretation of the MMC-dependent reduction in fork abundance is that it is due to inhibition of origin firing, but only a fraction of origins are inhibited. If so, one unanswered question is whether this inhibition is applied randomly or according to a specific feature. For example, the inhibited origins may reside in genomic domains undergoing DNA damage response (DDR). To address this question we asked whether we can detect DNA replication in the areas of chromatin engaged in DDR after MMC treatment.

### γH2AX is detected on nascent DNA after MMC treatment

**Figure 5:**
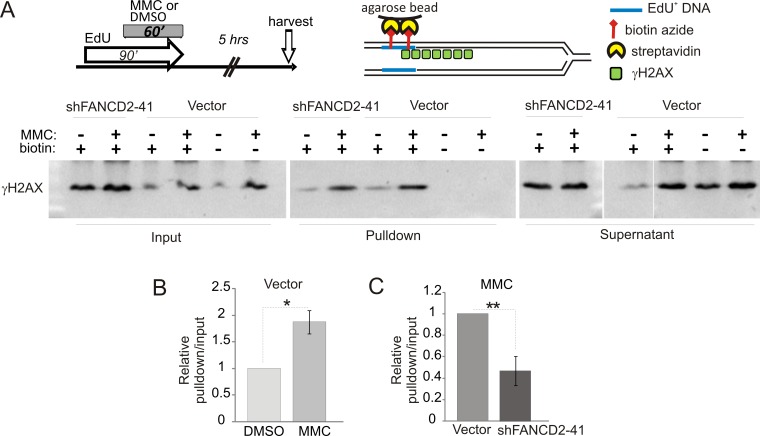
γH2AX is preferentially localized to DNA replicating after MMC **A**) An experimental design, a depiction of iPOND procedure, and a Western blot of iPOND samples probed with γH2AX antibody. Samples were generated as described in Materials and Methods, from GM639cc1 cells with empty vector lentiviral cassette (pLKO.1) or an shRNA against FANCD2. 5% of the lysate was removed before incubation with streptavidin beads and loaded in the input panel. No-biotin lanes are reactions that were mock-Clicked with DMSO (vehicle) instead of biotin-azide prior to incubation with streptavidin beads. 5% of supernatant from streptavidin bead step was loaded in Supernatant panels. **B**) A quantitation of γH2AX iPOND signals in GM639cc1 cells with an empty vector cassette. γH2AX signal intensity in Western blots typified by (B) was determined as described in Materials and Methods, and ratios of pulled-down to input γH2AX was determined. The pulldown/input ratios of DMSO-treated control obtained in two independent experiments were set to 1 unit (relative pulldown/input) and the ratios obtained for MMC-treated cells were expressed in these units, then averaged for the two experiments. **C**) A quantitation similar to (B) was performed on three independent experiments carried out with MMC-treated GM639cc1 cells with empty vector lentiviral cassette (pLKO.1) or an shRNA against FANCD2. Pulldown/input γH2AX signal ratios obtained for vector control were set as 1 unit and the ratio obtained for shFANCD2 cells was expressed in these units and averaged between experiments. Error bars in (C, D) are standard deviations and statistical significance was determined in t tests.

Serine 139 phosphorylation on H2AX (γH2AX) rapidly occurs at sites of DNA damage and over time it spreads bidirectionally, forming up to megabase-size chromatin domains [22, 23]. We showed that γH2AX is induced by our MMC treatment regimen (Figures 1, S2, S3). Thus, we now used iPOND (a DNA-mediated ChIP) to determine whether we can detect γH2AX on newly-replicated DNA in MMC-treated cells (Figure 5).

In the first series of experiments we labeled cells with EdU before and during MMC treatment for a total of 90 min, and harvested cells 5 hrs after MMC in order to maximize γH2AX domains. After fixation and permeabilization, biotin-azide was conjugated to EdU (Click-It reaction) in order to precipitate EdU-containing DNA and associated proteins with streptavidin beads (Figure 5A). As expected, MMC-dependent induction of γH2AX was clearly evident in input samples (Figure 5A, Input panel), and a fraction of this γH2AX was associated with EdU+ DNA in a biotin-dependent manner (Figure 5A, Pulldown panel). Importantly, in wild type cells a significantly higher fraction of total γH2AX was found on EdU+ DNA after MMC compared to DMSO (Figure 5B), suggesting that γH2AX was overrepresented in the vicinity of DNA that had replicated after MMC-induced damage. Accordingly, virtually no overrepresentation of γH2AX was detected on DNA that had replicated before it was subjected to MMC (Figure S5A).

In FANCD2-depleted cells, baseline and MMC-induced γH2AX levels were somewhat higher than in control, as expected (Figure 5A). However, this did not translate into a higher representation of γH2AX on EdU+ DNA (Figure 5C). This interesting result (see Discussion) cannot be explained by the reduced amount of EdU in DNA of FANCD2-cell samples, since the length and abundance of replication tracks are typically similar in FANCD2+ and FANCD2-depleted cells after MMC (Figures 3, 4, also Figure S5C). Overall, these findings indicate that new replication is not excluded from the areas of DNA damage response to MMC lesions, at least when it occurs immediately after MMC. In fact, DDR may be enhanced as a consequence of replication.

**Figure 6:**
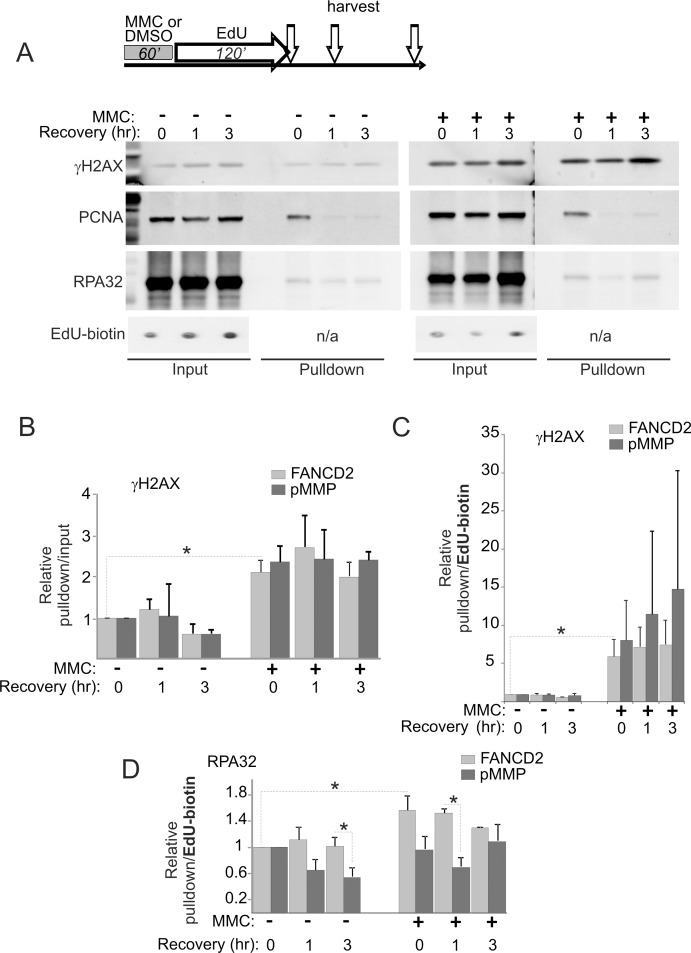
High level of γH2AX is recruited to DNA replicating immediately after MMC and remains largely unchanged for up to 3 hours **A**) An experimental design used throughout, and a representative example of Western blots performed on iPOND samples. Experiments were performed with PD20 cells stably expressing HA-FANCD2 transgene (from an integrated pMMP-based construct) or mock-expressing controls (with an integrated empty pMMP vector). Western blots show representative data obtained for PD20/ pMMP cells. 3% of total lysates were loaded in input lanes. All quantitations shown in (B-D) are means of two independent experiments and error bars are standard deviations. **B**) Pulldown/ input ratios for γH2AX were determined as described in Figure 5, expressed relative to DMSO-treated values at 0 hr recovery in each cell line, and averaged. **C**) γH2AX signals in pulldowns were normalized to EdU signals in inputs (see Materials and Methods for more detail), expressed relative to DMSO-treated values at 0 hr recovery in each cell line, and averaged. **D**) RPA32 signals were quantified and plotted in the same manner as γH2AX signals in (C). Asterisks denote p values determined in t tests in select pairwise comparisons. Note that in (B-D) only a representative subset of tests is shown and absence of asterisks does not mean that the difference did not reach statistical significance.

To confirm this conclusion, we asked whether we can detect γH2AX on DNA immediately after replication rather than hours later. We treated cells with MMC (or DMSO), then labeled with EdU for 2 hrs (Figure 6A). The experiments were done in PD20 cells as demanded by large cell numbers needed per iPOND sample. Samples were taken immediately after EdU or up to 3 hrs later. We verified that PCNA was associated with nascent DNA immediately after EdU pulse but not hours after, as expected for a component of a moving replisome (Figure 6A). In order to more extensively quantify iPOND results, we introduced measuring of EdU-biotin conjugate in iPOND samples by dot blotting with HRP-conjugated anti-biotin antibody (Figure S5B,C). Thus, levels of proteins in pull-downs could be quantified in two complementary ways: as a fraction of the total protein (with respect to their levels in inputs, i.e. pulldown/input), or relative to EdU-biotin (i.e. pulldown/EdU-biotin). The latter approach can give an estimate of density of the protein of interest on labeled DNA. Also, it is not affected by changes in total cellular levels of the protein of interest that may be triggered by MMC treatment.

In order to compare independent experiments, the protein level at time point 0 hr in untreated control in each experiment (normalized as described above), was set as baseline for each cell line (i.e. FANCD2-deficient and complemented cells), and the rest of the values within an experiment were expressed relative to this baseline. The data showed MMC-dependent enrichment of γH2AX on nascent DNA as early as 2 hrs after the drug (i.e. at the end of EdU labeling, Figure 6B). This sustained enrichment of γH2AX on nascent DNA after MMC was also clearly evident when it was quantified relative to EdU+ DNA in the samples, where in two independent experiments it ranged from 4 to 8-fold for FANCD2+ cells and from 4 to 15-fold for FANCD2-deficient cells (Figure 6C). This variability is likely due to the fact that experiments were performed independently and separately for each cell line, focusing on comparisons between MMC/DMSO conditions rather than between cell lines. Nevertheless, it is possible to conclude from these data that the density of γH2AX on EdU+ DNA is elevated after MMC. In addition, γH2AX to EdU ratios showed that while in DMSO-treated cells the modest amount of γH2AX on nascent DNA dropped as this DNA matured (by 3 hrs after labeling), high level of γH2AX on DNA replicated following MMC pulse remained unchanged for at least 3 hrs after replication.

We also monitored association of RPA32 with nascent DNA with and without MMC and/or FANCD2 (Figure 6D). In two independent experiments performed for each cell line we observed that MMC induced more RPA32 to associate with nascent DNA in FANCD2+ cells but not in FANCD2-deficient cells. This is consistent with the previous observation that FANCD2 facilitates RPA recruitment to DNA [18], and was also reproduced in independent iPOND experiments where we directly compared EdU-bound RPA32 in FANCD2-deficient or proficient, MMC-treated PD20 cells (data not shown).

**Figure 7:**
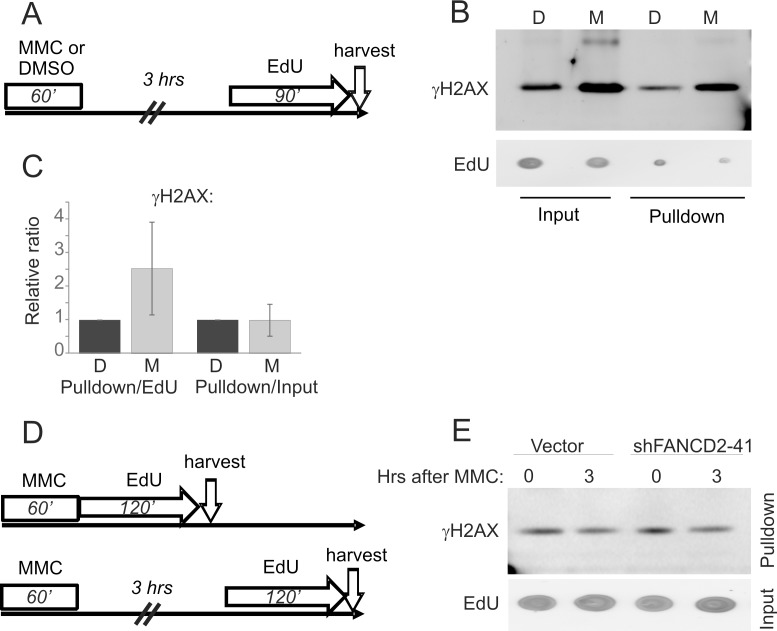
DNA that replicates 3-4 hours after MMC is not depleted of γH2AX **A-B**) An experimental design and a representative Western blot of iPOND samples generated with GM639cc1 cells expressing empty vector. 3% of total lysate used for streptavidin bead precipitation was loaded in input lanes and γH2AX and EdU levels were visualized and quantified. **C**) A quantitation of two independent iPOND experiments performed as shown in (A) with GM639cc1/vector control cells replicating 3 or 4 hrs after MMC. γH2AX signals in pulldowns were normalized to input EdU or input γH2AX, and plotted relative to DMSO-treated samples. Error bars are standard deviation. The difference between the first two lanes in (C) did not reach statistical significance. **D**) An experimental design for labeling DNA 0 or 3 hrs after MMC. **E**) A Western blot generated with iPOND samples of GM639cc1 control or shFANCD2-expressing cells, and showing γH2AX and EdU levels.

Finally, we asked whether DNA that replicates hours after MMC is also enriched with γH2AX. We treated cells with MMC, let γH2AX domains form for 3-4 hrs, and then labeled cells with EdU (Figure 7A, B). The density of γH2AX was higher on nascent DNA from MMC-treated cells compared to DMSO controls (Figure 7C, pulldown/ EdU bars), although the difference did not reach statistical significance across independent experiments. In addition, EdU-bound γH2AX was no longer overrepresented in the total γH2AX pool of the cell (Figure 7C, pulldown/input bars, compare with Figure 5B). A similar tendency was observed in FANCD2-depleted cells (data not shown). A direct comparison revealed that γH2AX density on DNA replicated 3 hr after MMC was lower than on DNA replicated immediately after MMC (Figure 7D-E).

Taken together, iPOND results indicate that, for several hours after MMC, DNA replication is not inhibited inside MMC-induced γH2AX domains. Moreover, DNA that replicates immediately after MMC captures and retains a disproportionately higher fraction of total γH2AX, which suggests that replication events early after the drug may in fact enhance DDR.

### FANCD2-depleted cells are less able to continuously replicate long stretches of DNA

The finding of enrichment of γH2AX on DNA that replicated early after MMC, prompted us to revisit the features of this replication. maRTA data indicate that 90-120 min labeling pulses used in iPOND experiments above, typically generate track length distributions with a mode of 60-80Kb and a maximum of 240Kb. We next used two or three labels (EdU, CldU, IdU) consecutively for a total labeling time of 90-120 min, to distinguish between ongoing versus completed replication tracks as containing, respectively, three or fewer labeled segments. The length distributions of these classes of tracks showed expected differences, although there was a substantial overlap between the distributions (Figure S6 shows examples of length distribution for a three-label regimen, and Figure 4E shows it for a two-label regimen). Because of this overlap, evaluating replication progression/termination based on sequential color incorporation was expected to be more sensitive than evaluating it by track length distributions. Failure of tracks to incorporate the last (or middle and last) label(s) is reasoned to be due to termination of replication. Tracks containing only the last label were not counted because we cannot determine whether they will or will not continue to replicate after the last pulse is over.

**Figure 8:**
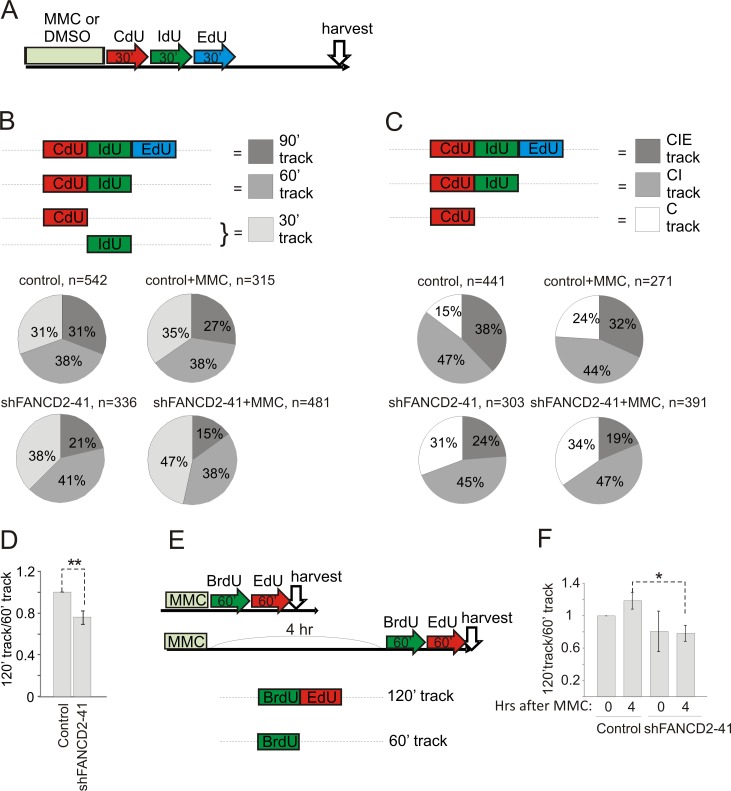
maRTA analysis shows that relative abundances of continuous long tracks generated during 90-120 min labeling window are reduced in FANCD2-depleted GM639cc1cells **A**) An experimental design involving consecutive pulse-labeling with three analogs after 25μM MMC (or DMSO). The experiment was performed with GM639cc1 cells expressing empty vector or FANCD2 shRNA. **B, C**) Track types counted and pie charts showing their relative abundances in each sample. 60′ and 30′ tracks have failed to incorporate third or second and third labels and thus are considered terminated. Note that in (C) exclusion of IdU only tracks enables to score *in-cis* replication behavior, i.e. elongation or termination within the domains that engaged in replication during the first label (CldU) pulse. Track totals for each sample are listed next to the respective pie charts. **D**) Tracks containing only first label, e.g. CldU (60′ tracks) or two consecutive labels, e.g. CldU-IdU (120′ tracks) after two pulses of labeling for 60 min each, were counted in DMSO-treated cells in two independent experiments with replicate samples. Average total track number counted per sample was 693 (min=270 and max=1297). Ratios of 120′ to 60′ tracks for each cell line were expressed relative to these ratios in empty vector control cells in each experiment, averaged, and plotted. **E**) An experimental design and types of tracks generated for the data presented in (F). **F**) Ratios of 120′ (e.g. BrdU-EdU) to 60′ (e.g. BrdU) tracks were determined in two independent experiments, expressed relative to these ratios in empty vector controls at 0hr after MMC in each experiment, averaged, and plotted. Average total number of tracks counted per sample was 559 (min=216, max=1233). Statistical significance was determined in t tests.

Experiments were performed on control and FANCD2-depleted GM639cc1 fibroblasts replicating immediately after MMC or 3-4 hrs later (Figure 8). In all experiments FANCD2-depleted cells showed fewer tracks that incorporated all labels compared to tracks that terminated before the pulse of second or third label. For example, in Figure 8A, B we scored relative frequencies of three-segmented CldU-IdU-EdU or CIE tracks (90′ tracks) versus one label only or two labels only tracks, respectively C or I (30′) and CI (60′). Cells were harvested for analysis 4 hrs after the labeling pulses to let nascent DNA maturate. FANCD2-depleted cells had proportionately fewer 90′ tracks and more 30′ tracks compared to controls, and this phenotype was observable with or without MMC in these cells (Figure 8B). This phenotype of FANCD2-depleted cells was also evident when we looked specifically at the mutual distribution of tracks that could represent sequential extension within one and the same replication domain, that is, C, CI, and CIE tracks (Figure 8C). Finally, this phenotype was reproduced with or without MMC treatment using a two-label protocol (Figure 8D-F), where in FANCD2-depleted cells tracks that incorporated first then second label (120′ tracks) were less abundant relative to tracks that incorporated only the first label (60′ tracks).

In summary, the labeling schemes designed to identify tracks of DNA replication that are extended uninterrupted for up to 120 min and up to 240Kb, made it possible to detect a statistically significant tendency toward fewer uninterrupted, long replication tracks in FANCD2-depleted cells, a phenotype that was not dependent on MMC. In addition, we detected a decrease in these uninterrupted tracks upon MMC treatment in wild type cells; this phenotype however, was too subtle to reach statistical significance.

## DISCUSSION

### Global effects of DNA crosslinkers on replication in vivo

Transient inhibition of DNA replication after DNA damage in eukaryotic cells has been known for years, however, it mechanistic dissection continues to reveal layers of complexity. It is currently understood that responses of the ongoing DNA replication to DNA damage include slowing of fork progression and inhibition of origin firing [24]. Slowing of forks was observed for camptothecin (CPT), X-rays, and UV, [25-28], and was argued to occur regardless of whether or not the forks collided with damage. The mechanism of such global slowing is not yet clear. Inhibition of new origin firing (replication initiation) is another genome-wide response to DNA damage or fork blockage, and can be observed after X rays, UV, hydroxyurea (HU) or aphidicolin [28-31]. Inhibition of origins that normally fire late in S phase is most readily observed, however, it is not known whether the late origins are the only ones targeted, and if so, how [24]. To complicate matters, origin inhibition may not apply uniformly throughout the genome, as replication domains already engaged in replication may fire extra origins if forks are slowed, while as yet unreplicated domains may delay to initiate replication [32, 33]. The mechanisms of this selectivity, and whether or not cell type or transformation status affect it, are not well understood [24]. Finally, an additional, novel mechanism that involves stalling specifically of newly originating replication forks at bulky DNA lesions proximal to origins, was observed recently in budding yeast [34].

One novel finding of this work is a direct demonstration that a dose of MMC that induces DNA damage response and eventually causes substantial cell death, triggers only a relatively mild, 50% or less reduction in fork abundance in S phase human cells. This phenotype is virtually identical in early passage primary cells and in immortalized human cells with an extensive history of passaging; and FANCD2 deficiency does not eliminate it. The reduction of fork abundance persists for hours after MMC and thus can explain the commonly seen profound MMC-dependent slowing of S phase, provided it is a compounding effect that continues to limit the number of forks active in any given window of time throughout S phase.

On the other hand, we detected no global reduction in fork progression rate after MMC in multiple treatment and sampling protocols, and regardless of FANCD2 status. A similar result was derived with crosslinkers such as CisPt (up to 330uM) and up to 20uM trioxalen/0.9J/ m2 UVA (data not shown). Interestingly, CisPt, psoralen/ UVA and MMC effects on fork progression have been previously reported in CHO and DT40 cells [35-37]. Recently, Huang et al [11] demonstrated local slowing followed by bypass by forks at trioxalen crosslinks. However, to our knowledge only one published study addressed effects of MMC on replication in human U2OS cells, demonstrating mild fork stalling, no fork rate reduction, and origin firing inhibition with 300μM (0.1mg/ ml)/1 hr MMC treatment regimen [28]. Utilizing a similar treatment and labeling scheme with 100μM MMC, we likewise show a very weak phenotype both in FANCD2-proficient and deficient cells that appears to be limited to new origin firing (for PD20 fibroblasts, Figure S4). It is possible that an organism-specific difference may account for the lack of fork progression phenotype in human versus non-human cells.

Cumulatively, our data suggest that reduction of fork abundance after MMC may be due to the persistent inhibition of initiation or establishment of processive forks at a fraction of origins. This raises two considerations that, to our knowledge, have not yet received attention. First, persistent reduction in the number of active forks implies that genomic replication as a defined program of sequential activation of megabase-sized spatio-temporal replication domains [38], may be profoundly disrupted by MMC damage. Second, it is not clear what determines which origins are inhibited, and whether a subset of origins within each replication domain or a subset of entire domains are prevented from replicating.

### DNA replication is not excluded from γH2AX-marked chromatin

One simplistic assumption may be that new replication is preferentially inhibited in the domains that contain lesions already recognized by DNA damage response (DDR) machinery. We addressed this hypothesis by asking whether after MMC newly replicated DNA is depleted of the DDR marker γH2AX. γH2AX formation is best studied during recognition and repair of DSBs, where it spreads over at least several hundred Kbps on either side of the lesion [39, 40], however, γH2AX also forms during UV-induced replication stress [41] or senescence [42]. Importantly, even smaller γH2AX domains (<200Kbp) can equal or exceed the size of one or more replicons (50-200Kbp).

We used nascent DNA-mediated immunoprecipitation, or iPOND [43, 44] to show that the density of γH2AX captured per unit of newly replicated (EdU+) DNA in MMC-treated cells was significantly higher than in untreated cells immediately after the drug and for up to several hours later, confirming induction of γH2AX by MMC. To evaluate whether or not γH2AX was preferentially associated with EdU+ DNA, we used the following reasoning. If γH2AX domains formed throughout the genome, and EdU labeled a random subset of these domains, then the amount of EdU-associated γH2AX should rise with the amount to total γH2AX while their ratio should remain constant regardless of MMC. If replication is selectively inhibited in γH2AX-enriched domains, then this ratio should be lower in MMC-treated compared to untreated cells. We found that this ratio was in fact higher for DNA that replicated immediately after MMC, i.e. γH2AX associated with EdU+ DNA was overrepresented in the total γH2AX pool. This overrepresentation of γH2AX was no longer evident on DNA replicating 3-4 hrs after MMC. This novel finding could mean that replication locally enhances development of γH2AX response. This agrees with the findings that replication may differentially affect recognition and repair of MMC damage [3]. Alternatively, replication early after MMC could be preferentially occurring in γH2AX domains.

γH2AX was shown to be upstream of FANCD2, recruiting it to chromatin after MMC [45]. Interestingly, we found that FANCD2 depletion reduced overrepresentation of γH2AX on DNA that replicated early after MMC. This effect was observed in FANCD2-depleted fibroblasts when levels of γH2AX were inspected 5 hrs after MMC, but was not obvious in FANCD2 mutant fibroblasts sampled 0 to 3 hrs after MMC, albeit the latter experiment was not designed to quantify γH2AX induction across different cell lines. It is nevertheless possible that FANCD2 may modulate maintenance of γH2AX response on replicated DNA, which does not contradict Bogliolo *et al* findings referenced above.

Overall our iPOND results indicate that γH2AX is certainly not depleted from DNA engaging in replication for at least 3-5 hrs after MMC, suggesting that replication is not inhibited in DDR-marked domains, at least in the transformed cells used in this study. From the length distribution of EdU tracks generated for iPOND measurements (60-80Kb mode, 240Kb max), we can conclude that a measurable fraction of ongoing DNA replication occurs within 60-240Kb of MMC-induced γH2AX domains. An increase in RPA32 presence on DNA replicating after MMC, which was evident in wild type but not in FANCD2 mutant cells, as expected [18], can also indicate that DNA was replicating in the proximity of ongoing DDR.

Overall, the advantage of our iPOND-based approach is that is offers higher resolution compared to the more common analysis based on foci immunofluorescence (IF), since it demonstrates physical association of γH2AX with DNA within less than 1Kb of actual site of EdU incorporation (≤1Kb is the size EdU DNA is sheared to for precipitation in iPOND), whereas colocalization of EdU and γH2AX in foci implies association anywhere within 250-300Kb.

### FANCD2 and replication after MMC

Since FANCD2 is required for repair of crosslinks and replication fork maintenance during replication stress, we expected its depletion to reveal strong replication fork phenotypes after MMC. Surprisingly, we only observed decreased prevalence of long replication runs covering up to 240Kb in the unperturbed as well as MMC-treated S phase cells. This phenotype is a novel though not unexpected observation. Long tracks sequentially labeled by three labels can represent extension by one fork and/ or a chain of replicon firing events happening one after another (a domino effect). Consequently, FANCD2-depleted cells may have less efficient elongation by one or more forks that are generated in a wave of time-staggered initiation events, and/or less efficient execution of initiation itself. Both scenarios fit within the context of reported FANCD2-dependent replication phenotypes, namely, its role in replication initiation [16] and in supporting fork stability when fork progression is partially or completely suppressed [17-19]. On the other hand, bypass of crosslinks by replication forks reported by Huang et al [11] was not dependent on FANCD2, which is consistent with our finding that FANCD2 depletion showed only mild MMC-dependent fork-related phenotypes.

In summary, our findings promote the view of crosslinks as lesions that restructure replication of the whole genome by systemically reducing replication fork abundance per unit of time and altering the chromatin context in which replication takes place. It remains to be determined whether crosslinker-induced reduction of replication fork abundance is random or exhibits spatio-temporal preferences that may differ between normal and transformed cells in ways that boost genomic stability and cellular survival.

## MATERIALS AND METHODS

### Cells and culture

SV40-transformed GM639 fibroblast cell line was obtained from the Coriell Institute Cell Repositories. GM639cc1 is a pNeoA derivative of GM639 [46-48]. The large T antigen is at least partially inactivated in this cell line since it does not support replication of SV40 origin-containing plasmids (JS, unpub.). PD20 SV40-transformed fibroblasts stably expressing HA-FANCD2, HA-FANCD2K561R, or empty vector pMMP are a gift of Dr. Taniguchi (FHCRC).

The low passage isogenic primary human dermal fibroblasts and keratinocytes were a gift of Dr. Galloway (FHCRC). All fibroblast cells were grown in Dulbecco Modified Minimal Essential Medium (DMEM) supplemented with L-glutamine, sodium pyruvate, 10% fetal bovine serum (Hyclone) and antibiotics, and keratinocytes were grown in Epilife media supplemented with HKGS (Life technologies) and antibiotics. Cells were kept in a humidified 5% CO, 37°C incubator.

### Drugs and Dyes

Stock solutions of 5-bromodeoxyuridine (BrdU; 10 mM in water, Sigma-Albrich), 5-iododeoxyuridine (IdU, 2mM in PBS, Sigma-Aldrich), 5-chlorodeoxyuridine (CldU, 10mM in water, Sigma-Aldrich), 5-ethynyldeoxyuridine (EdU, 10mM in DMSO, Life Technologies), mitomycin C (10mM in DMSO, Calbiochem) were stored at −20° C. CldU, BrdU, and IdU were used at concentrations of 50μM and EdU was used at 10μM.

### RNAi-mediated depletion of FANCD2

Short hairpin (sh) RNA pLKO.1-based constructs for depletion of FANCD2 were purchased from Open Biosystems (Thermo Scientific), clone IDs TRCN0000082841 (Cat. no. RHS3979-201909896 and TRCN0000082840 (RHS3979-202810221). The two shRNAs are identified by their last two digits (40 and 41) in the Figures, and where no number is provided, data obtained for both shRNAs were averaged. Depletions were carried out as described [47-49]. Controls included pLKO.1 empty vector, and two non-specific shRNAs: shNS, 5′-CTCCATATCGAACAGTTGG-3′ (stem sequence) and shNS2, 5′-CCTAAGGTTAAGTCGCCCTCG-3′.

### Western blotting and antibodies

To detect FANCD2, whole cell extracts prepared as described previously [47, 48] were resolved in pre-cast Tris-acetate gels (Life Technologies), transferred onto nitrocellulose membranes and probed with the α-FANCD2 antibody ab2187 (Abcam). Mouse α-CHK1 antibody was from Santa Cruz (Cat. No. sc-8408). Phosphorylation of CHK1 and CHK2 was analyzed with a Phospho-Chk1/2 Antibody Sampler Kit (Cell Signaling, Cat. No. 9931). Other antibodies were: α-PCNA Cat. No. sc-56 (Santa Cruz), α-RPA32 Cat. No. A300-244A (Bethyl Laboratories), α-γH2AX Cat. No. 05-636 (Millipore), α-Nucleolin Cat. No. 396400 (Life technologies). Biotin-conjugated EdU was visualized in dot blots with HRP-conjugated α-biotin antibody, Cat. No. 7075 (Cell Signaling). All proteins were visualized by ECL (Amersham or Thermo Scientific) and quantified using Storm Phosphoimager and ImageQuant software (Molecular Dynamics) or FluorChem Imager (Alpha Innotech), using manufacturer-supplied software. For presentation, images were saved in TIFF format, adjusted for brightness/contrast and cropped using Adobe Photoshop, and assembled into figures in CorelDraw. Brightness/contrast adjustments were made to entire images. In some cases an image of one and the same blot was cut and spliced to delete extraneous lanes or to change the order of lanes.

### Staining for BrdU and EdU incorporation and FACS

Staining for BrdU was done as described [47] with α-BrdU antibody MoBu-1 Cat. No. NA61 (Calbiochem), which demonstrates no cross-reactivity with EdU ([50] and data not shown). To stain for EdU, the above procedure was modified to include a Click-It step after DNA apurunization step, as follows. Cells were washed twice in 3% BSA in PBS. The following mix was prepared prior to use (in the order of addition to PBS): 20uM Alexa 647 azide; 11mM Na ascorbate; 2.2mM CuSO4 in PBS; added at 100μl per 2-5x105 cells and incubated with cells for 1 hr at room temperature in the dark. Cells were washed once in 3% BSA in PBS and blocked with PBS/3% BSA/3% Normal goat serum for 15 min at room temperature, before proceeding to staining against BrdU.

FACS data analysis and presentation were done with FACS express software (Phoenix Flow Systems).

### Microchannel fabrication, DNA fiber stretching and replication track analysis

These procedures were done as described [47, 51], with the following modifications to stain for EdU incorporation. Coverslips with apurinated DNA were washed once in 3%BSA in PBS and incubated for 30 min at room temperature with the following mix, made prior to use (in the order of addition to PBS): 20μM biotin-azide; 11mM Na ascorbate; 2.2mM CuSO4. Coverslips were washed once in PBS/3%BSA and blocked as described previously [47, 51]. Reagents to detect EdU-biotin were as follows, added in this order and together with reagents for IdU or CldU staining: Texas Red Neutravidin Cat. No. A2665, biotinylated anti-avidin D Cat. No. BA-0300 (Vector Laboratories), Texas Red Neutravidin. CldU was stained using Alexa 350 goat α-mouse antibody if Texas Red was used to visualize EdU. In some cases, the color assignments were reversed, and EdU was visualized with Alexa 350 Neutravidin (Cat. No. 11236), while CldU was visualized with Alexa 594 goat α-mouse antibody. All fluorophore-conjugated antibodies and neutravidins were from Life Technologies. Microscopy of stretched DNAs was performed on the Zeiss Axiovert microscope with a 40x objective.

### Microscopy image presentation

Visual scoring or measurement of features in microscopy images was done in sets of merged multicolor jpeg files using Zeiss AxioVision software. For presentation, images were adjusted for brightness/ contrast and cropped in Adobe Photoshop, and assembled into figures in CorelDraw. Adjustments were always done to entire images. In some cases, brightness/contrast of individual color channels was adjusted separately.

### iPOND

Immunoprecipitation of EdU-labeled DNA was performed essentially as described [52], with the following modifications. Aprotinin and leupeptin were added to the permeabilization buffer (0.25% triton X100 in PBS) to the final concentration of 1μg/ml ea. The buffer used for sonication and lysis of cells was as follows: 50mM Tris HCl pH8.0; 0.5%SDS; 0.1% Na dioxycholate; 0.25% TritonX100; 1μg/ml aprotinin; 1μg/ ml leupeptin. To measure EdU amounts in lysates, serial dilutions, typically from 0.2 to 0.02μl of each lysate (adjusted to 0.5μl each with PBS) were loaded onto a nitrocellulose membrane, dried for 1-2 hrs, blocked in 5% BSA in TBST, and incubated overnight with 1:150 dilution of HRP-conjugated α-biotin antibody Cat. No. 7075 (Cell Signaling) in 5%BSA, TBST overnight at 4°C, then washed and subjected to ECL and quantified using FluorChem Imager. Values obtained for 2-3 serial dilutions that were within linear range of signal were averaged. Specificity of staining was verified with equivalent amounts of control lysates of cells that had been incubated in Click-It mixture without biotin-azide. In some cases EdU was also measured in pulldowns. 1μl of pulldowns was diluted in 50μl of PBS and loaded as two-three 2-fold dilutions that were volume-adjusted to 0.5μl. Normalizing results by EdU levels in starting lysates or in pulldowns demonstrated similar results.

## SUPPLEMENTARY FIGURES


