# Supplementary material for: Mitomycin C reduces abundance of replication forks but not rates of fork progression in primary and transformed human cells
Source: Oncoscience. 2014 Jul 27;1(7):540–55. doi: 10.18632/oncoscience.70 (PMC4278321; doi:10.18632/oncoscience.70)
Supplement: Supplementary file 1 [file oncoscience-01-0540-s001.pdf]

Mitomycin C reduces abundance of replication forks but not rates of fork progression in primary and transformed human cells

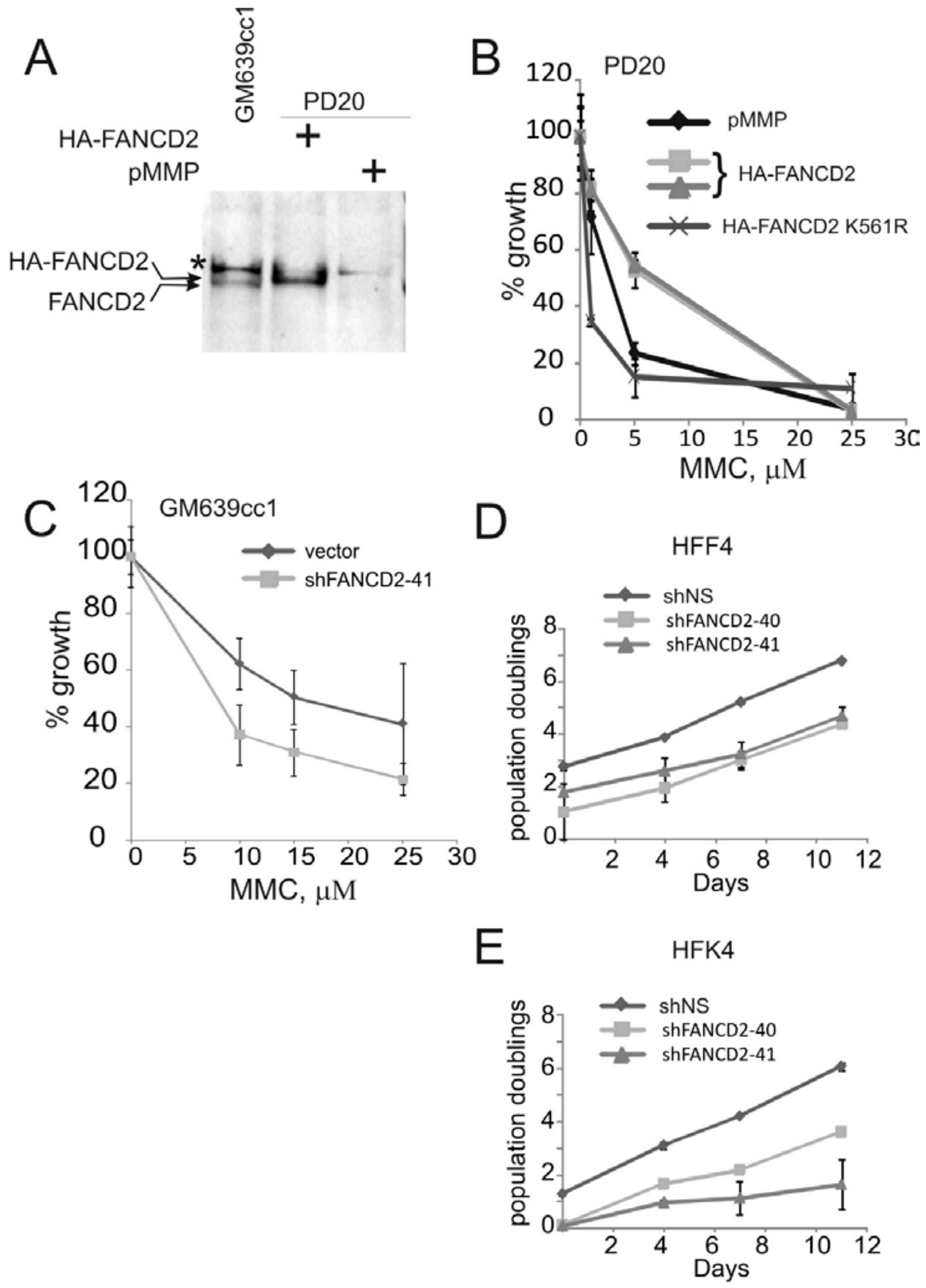

Figure S1. Suppressed cell growth and increased sensitivity to MMC in FANCD2-depleted primary and transformed human cells. **A)** A Western blot comparing levels of endogenous FANCD2 with the expression of HA-FANCD2 transgene in complemented PD20 cells. **B)** A growth curve comparing survival of vector-only and transgene-expressing PD20 cells after treatment with the indicated doses of

MMC for 1hr. Two independent clones derived from PD20 and stably expressing wild type HA-FANCD2 were used. HA-FANCD2 K561R is a clone expressing a ubiquitination mutant of FANCD2. Cell growth was quantified as described in Supplemental Materials and Methods 4 days after treatment. **C)** A growth curve comparing survival of vector-only and FANCD2 shRNA-expressing GM639cc1 cells after treatment with the indicated doses of MMC for 1hr. Cell growth was quantified as above on day 5 after treatment. **D, E)** Isogenic primary human fibroblast (HFF4) and keratinocyte (HFK4) cells expressing non-specific shRNA (shNS) or FANCD2 shRNAs (FANCD2-40 or FANCD2-41) were plated at 50,000 (HFF4) or 100,000 (HFK4) cells per well in 6-well plates. Cell number was counted in duplicate samples every two days to determine population doubling levels (PDLs) according to a formula PDL=ln(cell number on day N/cell number on day N+1)/0.693. PDLs were averaged and plotted. Error bars are standard deviations. Note that in some cases error bars are too small to show over markers.

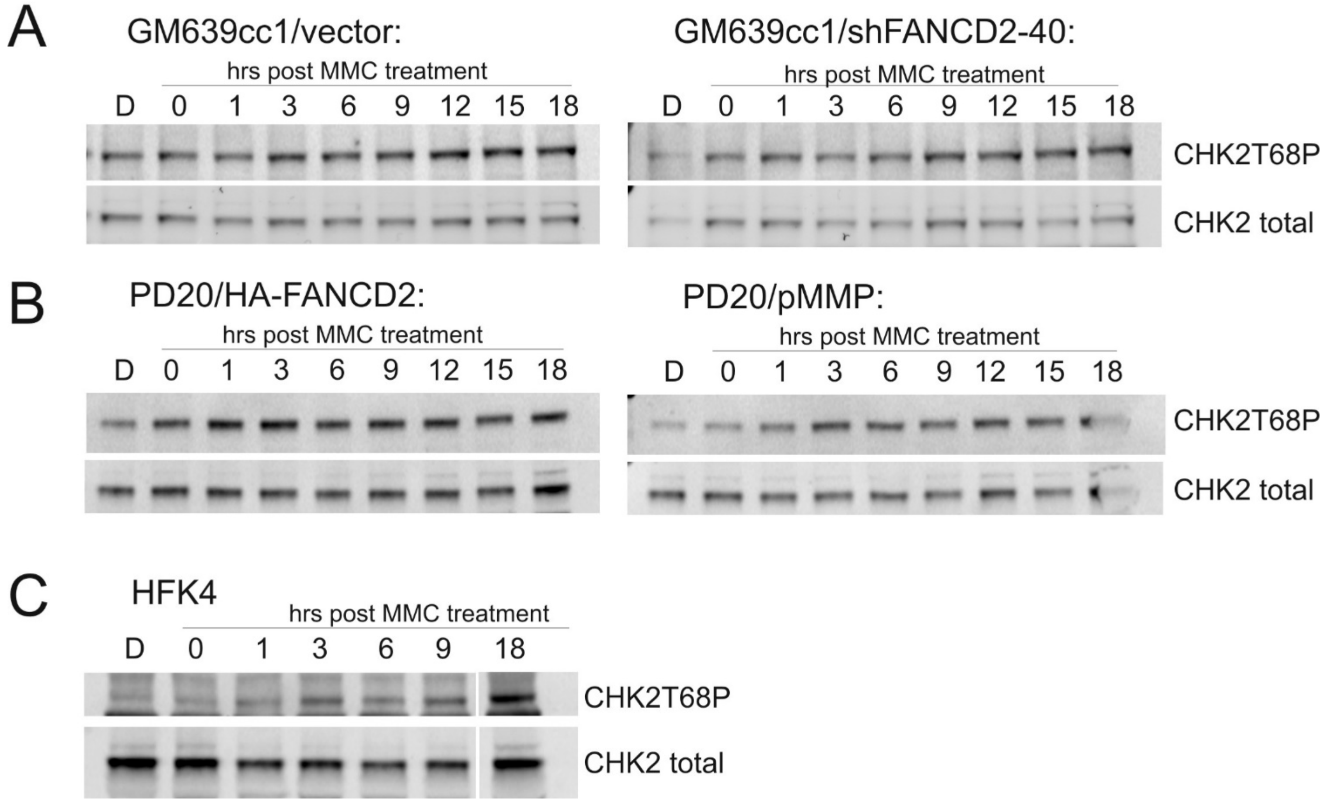

Figure S2. MMC-induced phosphorylation of CHK2 and γH2AX in human fibroblasts and keratinocytes with and without FANCD2. **A)** A Western blot of GM639cc1 fibroblasts expressing empty lentiviral vector pLKO (vector) or FANCD2 shRNA, treated with 25uM of MMC for 1hr (lane 0) and allowed to recover for indicated times before harvest. Lane D: DMSO (vehicle) treated control, harvested at time point 0. Threonine 68-phosphorylated (CHK2T68P) and total CHK2 were visualized. **B)** Western blots visualizing phospho-CHK2 and total CHK2 in PD20 fancd2-/- fibroblasts with and without HA-FANCD2 complementation. Cells were allowed to recover for up to 18hrs after MMC treatment (25uM/1hr). **C)** A Western blot visualizing phospho-CHK2 and total CHK2 in HFK4 keratinocytes treated with MMC (25uM/1hr) and allowed to recover for indicated times before harvest. Lane designations as in (A).

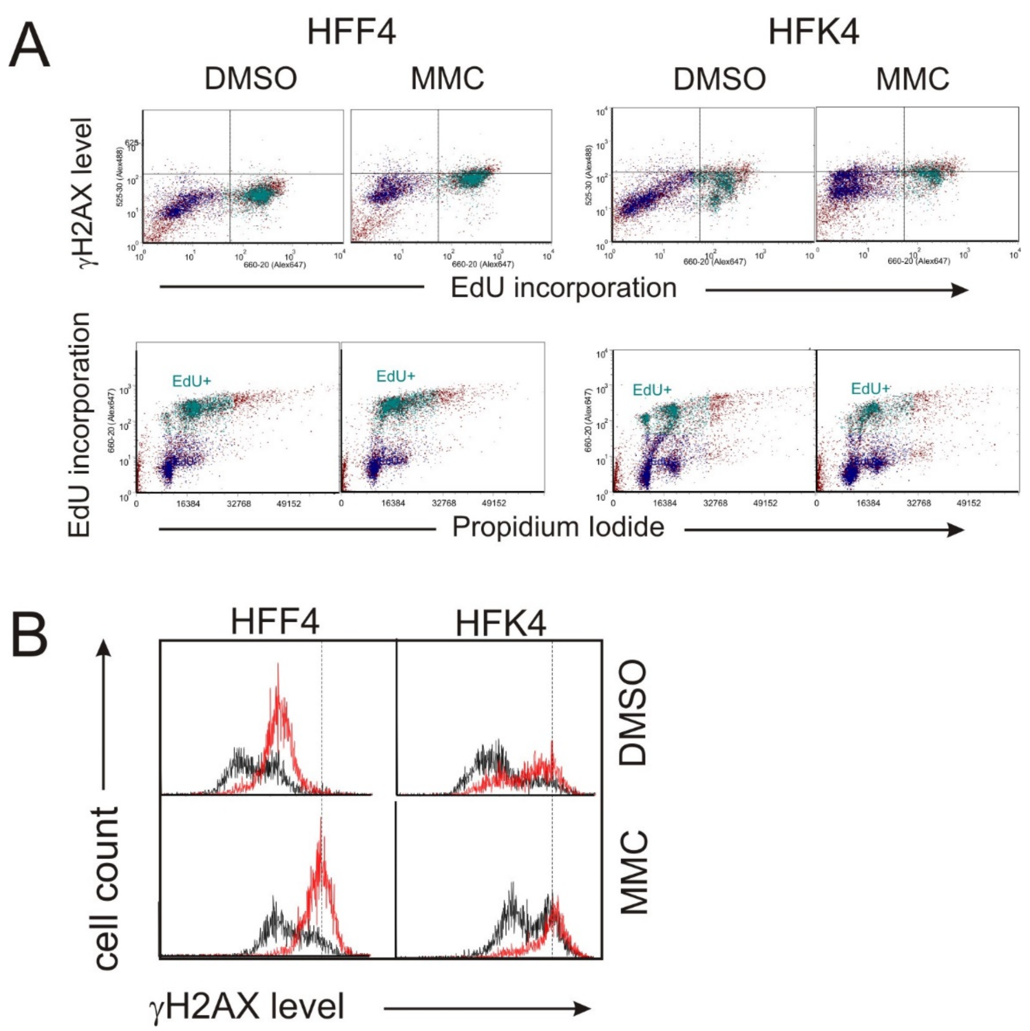

Figure S3. Flow-cytometric analyses of effects of MMC treatment on cell cycle progression and γH2AX expression in isogenic HFF4 and HFK4 cells. **A)** Examples of dot plots of flow cytometric data of cells labeled with EdU for 1hr, then treated with MMC (25uM/1hr) or DMSO (vehicle) and harvested 5.5hr after treatment. Cells were stained for EdU incorporation and γH2AX levels. Upper panels: Y axis is γH2AX level, X axis is EdU incorporation level. Quadrants specify positions of four possible cell sub-populations as defined by their EdU and γH2AX levels. Lower left and lower right quadrants are, respectively, EdU-negative (non S phase) and EdU-positive (S phase) cells with background γH2AX staining levels (as defined by staining of DMSO-treated cells). Lower panels: Dot plots of the same data

as in the upper panels, plotted by DNA content (Propidium Iodide staining), X axis, versus EdU incorporation, Y axis. Green coloring highlights EdU+ cells in all panels. **B)** Flow cytometric data from a representative experiment, showing γH2AX levels in cell populations 5.5 hrs after MMC (25uM/1hr) or DMSO treatment. Black profiles are EdU-negative populations and red profiles are EdU-positive populations.

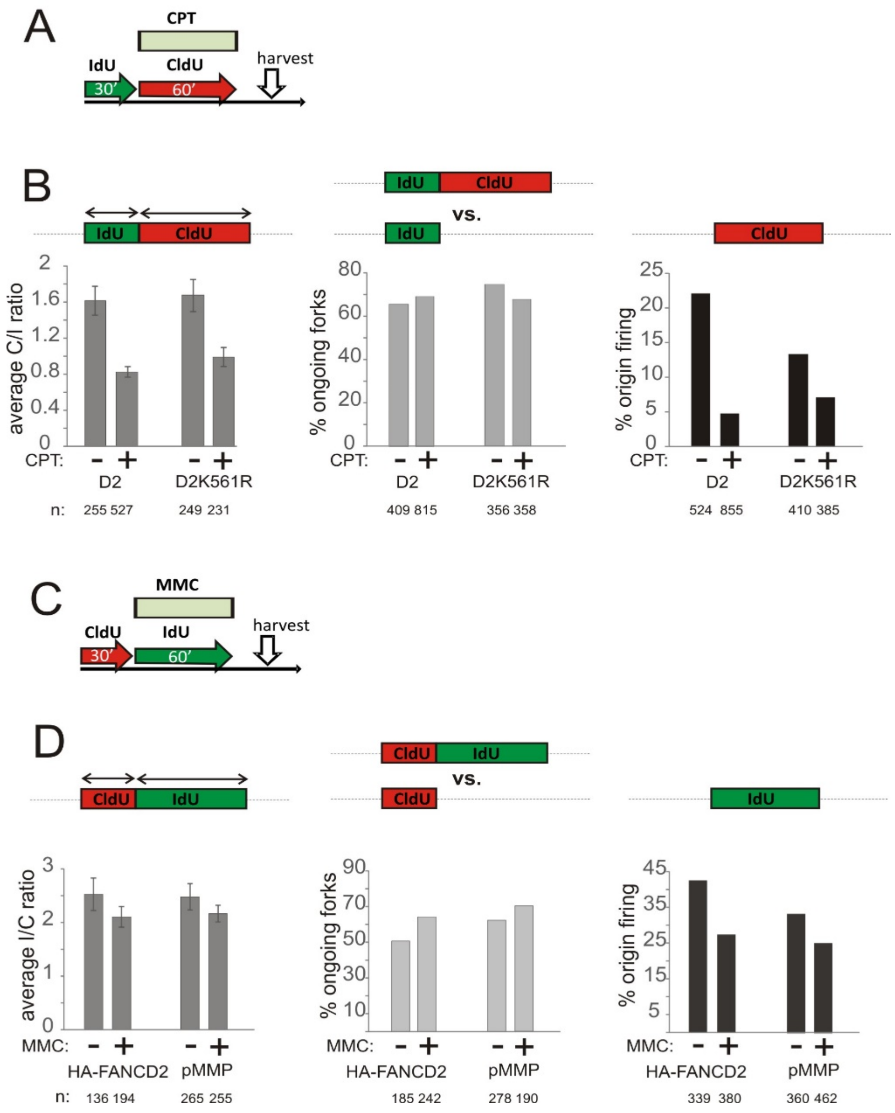

Figure S4. Comparison of maRTA analyses of effects of CPT and MMC on replication parameters in PD20 cells expressing empty vector or the HA-FANCD2 transgene. **A)** An experimental design. Cells were treated with 16μM CPT and harvested 30 min after incubation with the second label and the drug. **B)** Quantitations of replication parameters in PD20 cells expressing wild type or ubiquitination (K561R) mutant *FANCD2* transgenes. Left panel: relative ratios of lengths of CldU to IdU segments in two-segmented tracks representing ongoing forks. Ratios were averaged and plotted. Error bars are 95% confidence intervals of the means. Center panel: ongoing fork fraction defined as a proportion of two-segmented, IdU-CldU tracks among all tracks containing the first label, IdU. Right

panel: new origin firing frequency defined as a proportion of second label, CdU, only tracks among all tracks. In B through D, numbers of tracks that were analyzed in each sample to generate the plotted values are shown below the bar graphs. **C)** An experimental design. Cells were treated with 100μM MMC and harvested 30 min after incubation with the second label and the drug. **D)** Quantitations of replication parameters in PD20 cells expressing wild type *FANCD2* transgene or empty vector pMMP. Data presentation in bar graph panels is as described for (B), except CldU is the first label and IdU is the second label.

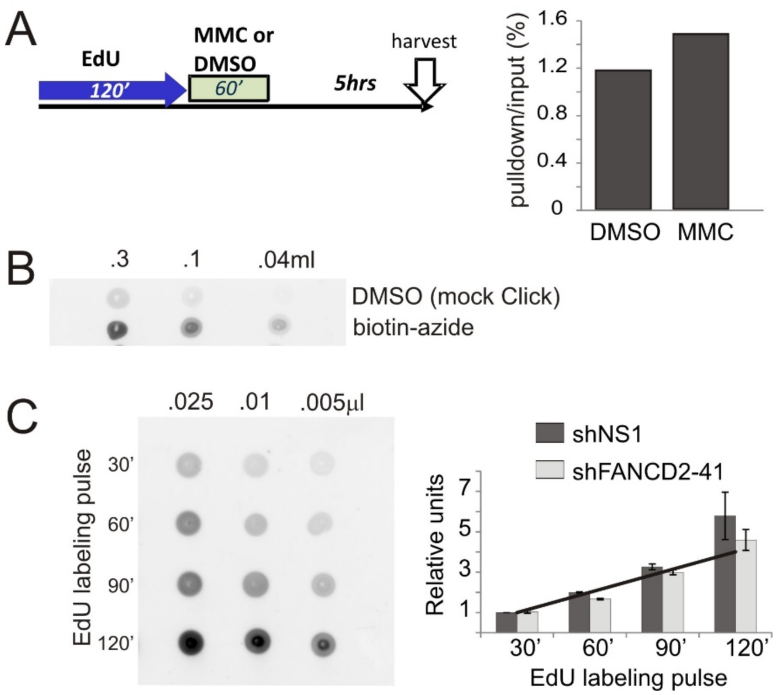

Figure S5. iPOND characterization of association of γH2AX with replicating DNA and establishment of EdU dot blot approach. All experiments were performed with GM639cc1 cells expressing empty vector or FANCD2 shRNA, and MMC concentration was 25uM. **A)** An experimental design and quantified data obtained in order to evaluate the fraction of γH2AX associated with EdU+ DNA in MMC-treated versus DMSO-treated cells if replication preceded the treatment. Pulldown/input % values were determined as follows: (protein signal in pulldown / protein signal in input) x fraction of total input loaded. **B)** Sonicated extracts from EdU-labeled cells were “clicked” to biotin-azide or DMSO (as a vehicle control), diluted with PBS, loaded onto nitrocellulose membranes and incubated with HRP-conjugated anti-biotin antibody as described in Materials and Methods. **C)** Extracts of cells labeled with EdU for 30, 60, etc. minutes were “clicked” with biotin-azide, diluted and dot blotted as in (B). EdU was quantified to determine the dilutions/labeling pulses within the linear range of EdU detection.

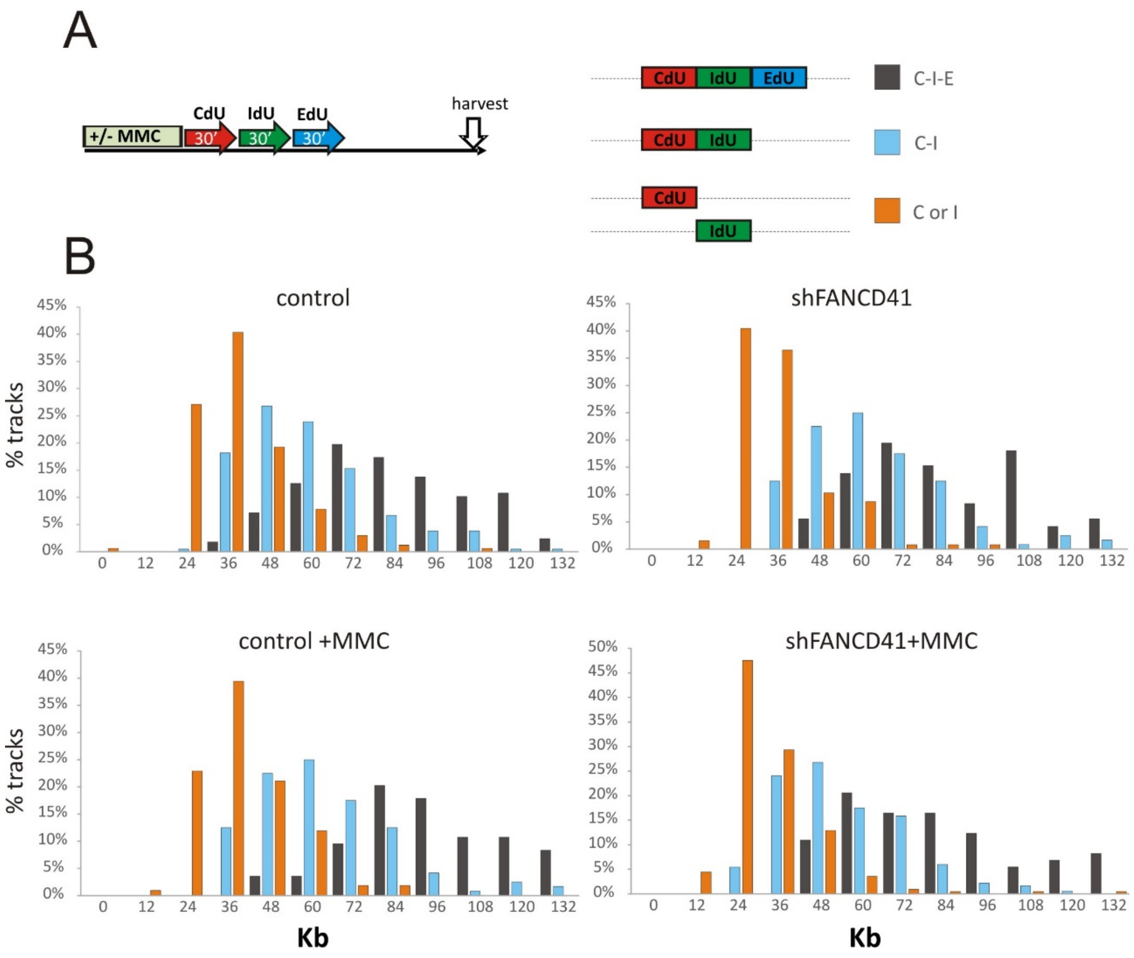

Figure S6. Length distributions of replication tracks generated by three consecutive pulses of three labels, and analyzed by maRTA. The experiment was performed in GM639cc1 cells expressing an empty vector or FANCD2 shRNA. **A)** An experimental design and types of tracks scored. **B)** Frequency distribution graphs of lengths of each track type (as specified in (A)) in the four samples analyzed. X axes values are binned values of track lengths in Kb (1μm=3.9Kb [1]), e.g. the bars corresponding to the value 24Kb in graphs are percentages of tracks with lengths between 12 and 24Kb.

Supplemental Materials and Methods

Population growth assay

Growth of cells after MMC treatment was performed as described previously [2], using crystal violet extraction method to evaluate cell abundance.

Supplemental References

1. Sidorova JM, Li N, Schwartz DC, Folch A and Monnat RJ, Jr. Microfluidic-assisted analysis of replicating DNA molecules. Nature Protocols. 2009; 4(6):849-861.
2. Chen C, Taniguchi T and D'Andrea A. The Fanconi anemia (FA) pathway confers glioma resistance to DNA alkylating agents. J Mol Med. 2007; 85(5):497-509.
